# Treatment of Colorectal Cancer Using a Combination of Liposomal Irinotecan (Irinophore C™) and 5-Fluorouracil

**DOI:** 10.1371/journal.pone.0062349

**Published:** 2013-04-23

**Authors:** Jennifer I. Hare, Robert W. Neijzen, Malathi Anantha, Nancy Dos Santos, Natashia Harasym, Murray S. Webb, Theresa M. Allen, Marcel B. Bally, Dawn N. Waterhouse

**Affiliations:** 1 Department of Pharmacology, University of Alberta, Edmonton, Canada; 2 Department of Pharmaceutical Science, Universiteit Utrecht, Utrecht, Netherlands; 3 Experimental Therapeutics, BC Cancer Agency, Vancouver, Canada; 4 Centre for Drug Research and Development, Vancouver, Canada; 5 Department of Pathology and Laboratory Medicine, University of British Columbia, Vancouver, Canada; 6 Faculty of Pharmaceutical Sciences, University of British Columbia, Vancouver, Canada; Aristotle University of Thessaloniki, Greece

## Abstract

**Purpose:**

To investigate the use of liposomal irinotecan (Irinophore C™) plus or minus 5-fluorouracil (5-FU) for the treatment of colorectal cancer.

**Experimental Design:**

The effect of irinotecan (IRI) and/or 5-FU exposure times on cytotoxicity was assessed *in vitro* against HT-29 or LS174T human colon carcinoma cells. The pharmacokinetics and biodistribution of Irinophore C™ (IrC™) and 5-FU, administered alone or in combination, were compared *in vivo*. A subcutaneous model of HT-29 human colorectal cancer in Rag2-M mice was utilized to assess the efficacy of IrC™ alone, and in combination with 5-FU.

**Results:**

The cytotoxicity of IRI and 5-FU were strongly dependent on exposure time. Synergistic interactions were observed following prolonged exposure to IRI/5-FU combinations. Pharmacokinetics/biodistribution studies demonstrated that the 5-FU elimination rate was decreased significantly when 5-FU was co-administered intravenously with IrC™, versus alone. Significant decreases in 5-FU elimination were also observed in plasma, with an associated increase of 5-FU in some tissues when 5-FU was given by intraperitoneal injection and IrC™ was given intravenously. The elimination of IrC™ was not significantly different when administered alone or in combination with 5-FU. Therapeutic studies demonstrated that single agent IrC™ was significantly more effective than the combination of IRI/5-FU; surprisingly, IrC™/5-FU combinations were no more effective than IrC™ alone. The administration of combinations of 5-FU (16 mg/kg) and IrC™ (60 mg IRI/kg) showed increased toxicity when compared to IrC™ alone. Treatment with IrC™ alone (60 mg IRI/kg) delayed the time required for a 5-fold increase in initial tumor volume to day 49, compared to day 23 for controls. When IrC™ (40 mg IRI/kg) was used in combination with 5-FU (16 mg/kg), the time to increase tumor volume 5-fold was 43 days, which was comparable to that achieved when using IrC™ alone (40 mg IRI/kg).

**Conclusions:**

Single agent IrC™ was well tolerated and has significant therapeutic potential. IrC™ may be a suitable replacement for IRI treatment, but its use with free 5-FU is complicated by IrC™-engendered changes in 5-FU pharmacokinetics/biodistribution which are associated with increased toxicity when using the combination.

## Introduction

Colorectal cancer (CRC) is a leading cause of cancer death worldwide [1], [2], [3]; in the United States, CRC is the third most common cause of cancer death and the third most commonly diagnosed cancer, with nearly 150,000 new cases estimated to be diagnosed in 2013 [4], [5]. The chemotherapeutic drug irinotecan (IRI) is used in several first-line CRC treatment regimens. Although IRI itself is active, non-specific plasma, liver, gastrointestinal (GI), and tumor carboxylesterases [6], [7], [8] can metabolize IRI to SN-38, which is 100- to 1,000-fold more potent when tested using *in vitro* assays [9], [10]. Gut carboxylesterases (CE) generate high local concentrations of SN-38 [11], [12]. This conversion can be associated with therapeutic activity, however, it has also been linked to the intestinal damage that is responsible for much of IRI’s adverse GI toxicity [13], [14], [15]. A secondary drawback to the use of IRI and SN-38 is the pH-dependent hydrolytic conversion from an active lactone form, at acidic pH, to an inactive carboxylate form, at physiological pH, which limits the dose of active drug that reaches the target [16], [17], [18], [19]. Some of the adverse toxicities and CE-mediated conversion of IRI can be ameliorated through the use of drug delivery systems [20], [21], [22], [23], [24], [25]. Irinophore C™ (IrC™) is a formulation of IRI encapsulated in unilamellar, 1,2-distearoyl-sn-glycero-3-phosphatidylcholine (DSPC)/cholesterol liposomes (100 nm diameter) containing an acidic aqueous interior of unbuffered CuSO_4_. IRI is entrapped in the acidic aqueous interior of the liposomes when a pH gradient is generated in the presence of the divalent metal ionophore A23187, which is required for the stability and maintenance of the pH gradient [21], [26]. The combination of the ionophore-generated pH gradient, together with the presence of encapsulated Cu^2+^, results in excellent drug retention properties for the formulation *in vivo* [26], [27], [28].

In preclinical studies, IrC™ demonstrated that the activity of IRI can be increased significantly in a wide range of tumor models [21], [26], [29], [30], with an improved safety profile relative to the free drug [21]. The increase in therapeutic index for IrC™, versus IRI, is thought to be due to several factors: i) maintenance of IRI in its active lactone form for extended time periods [21], [27], [30]; ii) increased delivery of IRI to sites of tumor growth [21]; iii) prolonged systemic exposure to the active lactone form of SN-38 [21]; and iv) the existence of an anti-vascular activity that is not observed following bolus administration of free IRI [29], [31]. We hypothesized that the therapeutic impact of IrC™ will be most significant when it is used as part of a drug combination – for example, in the chemotherapy regimen FOLFIRI (leucovorin, 5-fluorouracil (5-FU), and IRI), where IrC™ would be substituted for free IRI. This hypothesis was tested in a pre-clinical setting here, where combinations of IrC™/5-FU and IRI/5-FU were evaluated in a murine model of CRC. To our knowledge, this is the first research investigating the use of liposomal IRI formulations, including IrC™, in combination with 5-FU for the treatment of CRC. The therapeutic results, surprisingly, demonstrated that the efficacy of this combination was no better than that achieved with IrC™ monotherapy. Additionally, in the model used here, there was an unexpected increase in the toxicity of the drug combination, which required a dose reduction of IrC™ to a level that was far less active than a higher dose of IrC™, but could be administered safely when used as a single agent.

## Materials and Methods

### Materials

DSPC and cholesterol were obtained from Avanti Polar Lipids (Alabaster, Alabama, US). [^3^H]Cholesteryl hexadecylether (CHE) was purchased from PerkinElmer (Waltham, Massachusetts, US). [^14^C]5-FU was purchased from Moravek Biochemicals (Brea, California, US). A23187 was purchased from Sigma-Aldrich (Oakville, Ontario, CA). Saline, 5% dextrose in water (D5W), irinotecan (Camptosar, Sandoz), and 5-FU (Alfa Aesar) were obtained from the BC Cancer Agency (Vancouver, British Columbia, CA). The alamarBlue reagent, fetal bovine serum (FBS), L-glutamine, and sodium bicarbonate were purchased from Invitrogen (Burlington, Ontario, CA). Eagle’s minimum essential medium (MEM) with Earle’s balanced salt solution (BSS), McCoy’s 5A medium, Hank’s BSS (HBSS), non-essential amino acids, sodium pyruvate, and penicillin/streptomycin were purchased from StemCell Technologies (Vancouver, British Columbia, CA). All other chemicals were of analytical grade.

### Cell Culture

The human colorectal cell lines LS174T and HT-29 were obtained from ATCC (Manassas, Virginia, US). Stock cells lines were maintained in the absence of penicillin and streptomycin, and were screened for *Mycoplasma* prior to preparing a stock of cells that was frozen for use in experiments. Cells were re-suspended in freezing media (10% (vol/vol; v/v) dimethyl sulfoxide in FBS) and slowly frozen in Nalgene 1°C freezing containers (Rochester, New York, US) containing 100% isopropanol at −80°C for 24 h before storage in liquid nitrogen. Frozen cells were quickly thawed at 37°C, centrifuged to remove freezing media, plated and passaged twice before use in experiments. LS174T cells were cultured in Eagle’s MEM with Earle’s BSS supplemented with 2 mM L-glutamine, 1 mM sodium pyruvate, 0.1 mM non-essential amino acids, 1.5 g/L sodium bicarbonate, 1% (v/v) penicillin/streptomycin, and 10% (v/v) FBS, at 37°C in a 5% CO_2_ environment. HT-29 cells were cultured in modified McCoy’s 5A medium supplemented with 1.5 mM L-glutamine, 2.2 g/L sodium bicarbonate, 1% (v/v) penicillin/streptomycin, and 10% (v/v) FBS, at 37°C in a 5% CO_2_ environment.

### Cytotoxicity Assays

The viability of human CRC cell lines following exposure to different concentrations of IRI and/or 5-FU was determined using the alamarBlue assay [32], [33]. Cells (LS174T, 10,000 cells/well; HT-29, 5,000 cells/well) were seeded in flat-bottomed 96-well plates. After cell adherence had occurred, increasing concentrations of IRI or 5-FU were added to cells for 1–72 h, with drug washout as required at the indicated time point. In experiments to determine the time dependency of the exposure of the cells to drug combinations, HT-29 cells were exposed to IRI/5-FU at a 1∶1 molar ratio for 1–48 h, with drug washout as required at the indicated time point(s). For all experiments, cell viability was assessed at 72 h after the initiation of drug exposure. The alamarBlue reagent was added to each well at a 1∶10 dilution, and the cells were incubated for an additional 4–8 h before fluorescence was measured. For viability data, the fraction affected (FA) was a measure of the alamarBlue fluorescence normalized to the fluorescence of controls: a no cells control defining the 100% affect level and a drug-free control defining the 0% affect level. Interactions between IRI/5-FU when used in combination *in vitro* were determined on the basis of a single assay endpoint (alamarBlue viability assay, above), and the results were analyzed via the Median-Effect Principle [34], as estimated with CompuSyn software (ComboSyn, Inc.; Paramus, New Jersey, US) [35]. For each exposure time, dose-response curves were generated for the agents, alone and in combination, and, subsequently, combination index (CI) values were estimated at various affect levels (defined as fraction affected). A CI value of 0.8–1.2 represents an additive interaction, less than 0.8 represents a synergistic interaction, and greater than 1.2 represents an antagonistic interaction.

### Preparation of Irinophore C™

IrC™ was prepared as described by Ramsay *et al*. [26]. DSPC:cholesterol (55∶45 mol %) liposomes were prepared as previously outlined [36], [37], using trace amounts of the non-metabolizable, non-exchangeable lipid tracer [^3^H]CHE [38]. The thin lipid film was hydrated with a 300 mM CuSO_4_ solution at 65°C, the resulting lipid vesicles were subjected to 5 cycles of freeze-and-thaw, and the liposomes were extruded to a diameter of ∼100 nm. Unencapsulated CuSO_4_ was removed via chromatography using a Sephadex G-50 column, equilibrated with SHE buffer (300 mM sucrose, 20 mM HEPES, 15 mM EDTA; pH = 7.5). Liposomes were incubated with 0.5 µg A23187/mg total lipid for 30 min at 60°C. IRI was added to the liposomes at a molar drug-to-lipid ratio of 0.2∶1, and the mixture was incubated at 50°C for 1 h. Unencapsulated drug was removed via chromatography on a Sephadex G-50 column, equilibrated with PBS (pH = 7.4), and drug loading efficiency was determined after measuring IRI absorbance at 370 nm. When required, IrC™ was concentrated at 3,000×*g* using centrifugal filter tubes (molecular weight cutoff 100 kDa).

### Animals and Ethics Statement

All *in vivo* experiments were conducted utilizing 129S6/SvEvTac-*_Rag2_^tm1Fwa^* (Rag2-M) mice obtained from the BC Cancer Agency’s Animal Resource Centre at the Vancouver Research Centre (Vancouver, British Columbia, CA). The studies were conducted in accordance with the Canadian Council on Animal Care Guidelines with oversight from the University of British Columbia’s Animal Care Committee (protocols A10-0171 and A10-0206). Mice were housed under standard conditions with enrichment, with access to food and water *ad libitum*.

### Pharmacokinetics and Biodistribution

Experiments were completed to determine whether the simultaneous administration of IrC™/5-FU, via intravenous (i.v.) or intraperitoneal (i.p.) injection, altered the PK/BD properties of either agent. Male Rag2-M mice (8–10 weeks old; 4 mice per time point) received i.v. injections, via the lateral tail vein, of [^3^H]CHE-labeled IrC™ (40 mg IRI/kg), [^14^C]5-FU (40 mg/kg), or co-administered [^3^H]CHE-labeled IrC™ and [^14^C]5-FU (40 mg IRI/kg and 40 mg/kg, respectively), in a total injection volume of 0.2 mL. The dose of 5-FU selected for these studies was based on previous experiments demonstrating that this dose was well tolerated when given on a once per week (Q7D) dosing schedule, comparable to that used for IrC™ (results not shown). At various time points post-injection, mice were euthanized via CO_2_ asphyxiation. Blood was immediately collected via cardiac puncture, and centrifuged to separate the plasma. Organs were harvested and divided into 2 pieces; half of the plasma or organ was prepared for liquid scintillation counting (LSC) to determine the level of associated radioactivity (lipid and 5-FU), while the other half was processed for HPLC analysis to determine IRI and SN-38 levels. To limit conversion between the lactone and carboxylate forms, plasma samples and organs were kept on ice and transferred to −70°C within 1 h of collection.

A separate study was conducted to determine whether the PK/BD properties of 5-FU, administered QD×2 via i.p. injection, were affected by co-administration with IrC™ at a dose of 60 mg IRI/kg. This dosing was comparable to that used in the efficacy studies described below. The experiment was completed using male Rag2-M mice (7–10 weeks old; 3 mice per time point). Mice were injected i.p. with 16 mg/kg 5-FU on days 1 and 2, with or without co-administration of IrC™ (60 mg IRI/kg) via i.v. injection on day 1 at 2 hours after the injection of 5-FU. When repeated doses of 5-FU were administered, the final dose contained [^14^C]5-FU as a label to trace 5-FU levels. At various time points post-injection, mice were euthanized via CO_2_ asphyxiation. Blood was immediately collected via cardiac puncture, and centrifuged to separate the plasma. Organs were harvested and stored on ice, and transferred to −70°C within 1 h of collection. Samples were prepared for LSC for measurement of the associated radioactivity.

In preparation for LSC, tissue homogenates (10% weight/volume) were prepared in saline using a Polytron homogenizer (Brinkmann Instruments; Rexdale, Ontario, CA), and 0.5 mL of each homogenate was then digested in 0.5 mL of Solvable (DuPont Canada; Mississauga, Ontario, CA) for 1 h at 50°C. After cooling to room temperature, samples were decolorized by the addition of 0.2 mL of 30% H_2_O_2_. These samples were then incubated overnight at 4°C to prevent excessive foaming. Scintillation cocktail was added to samples, and following dark-equilibration the radioactivity ([^3^H]CHE and [^14^C]5-FU) associated with the plasma and organs was quantitated via LSC.

In preparation for HPLC analysis, the second half of each organ was homogenized in ice-cold water. Drug and metabolites were extracted from the homogenate using ice-cold acetonitrile/methanol (1∶1 v/v) solution, and centrifugation at 14,000×*g* for 15 min to precipitate proteins. The supernatant was collected, and the concentrations of IRI (lactone and carboxylate forms) and SN-38 (lactone and carboxylate forms) in the organ supernatant and plasma samples were determined via HPLC. HPLC separation of IRI and SN-38 lactone and carboxylate forms was performed using a 250×4.6 mm C18 Symmetryshield column and C18 Symmetryshield guard column (Waters; Mississauga, Ontario, CA). Gradient elution was used with mobile phase A, composed of 75 mM ammonium acetate and 7.5 mM tetrabutylammonium bromide, adjusted to pH 6.4 with glacial acetic acid (Fisher Scientific; Nepean, Ontario, CA), and mobile phase B, composed of acetonitrile. Gradient profile was as follows: time = 0 min: 78% A:22% B, time = 10 min: 64% A:36% B, time = 12 min: 78% A:22% B, time = 20 min: 78% A:22% B. A 0.01 mL sample was injected onto the column (column temperature of 35°C) and eluted at a flow rate of 1 mL/min. The lactone and carboxylate forms of both IRI and SN-38 were detected using a Waters 2475 multi-wavelength fluorescence detector (Waters; Mississauga, Ontario, CA), set with time program events of λex = 370 nm, λem = 425 nm between 0 and 12.5 min for the IRI lactone and IRI carboxylate, and λex = 370 nm, λem = 535 nm between 12.5 and 20 min for the SN-38 lactone and SN-38 carboxylate. Prior to injection, all samples were maintained at 4°C to reduce conversion between the lactone and carboxylate forms of IRI or SN-38. Standard curves of the IRI lactone and SN-38 lactone were prepared by serial dilutions in a 2∶1:1 sodium acetate (100 mM):methanol:acetonitrile (pH 4.0) buffer. For the IRI carboxylate and SN-38 carboxylate, serial dilutions were prepared in a 2∶1:1 sodium borate (100 mM):methanol:acetonitrile (pH 9.0) buffer. The limit of quantitation for IRI and SN-38 lactone and carboxylate forms was 10 ng/mL. Plasma and tissue AUC values were calculated from concentration versus time curves (mean +/− standard deviation) using GraphPad Prism 5.00 software (GraphPad Software; La Jolla, California, US). Statistical significance for the PK/BD study was calculated via two-way analysis of variance with Bonferroni post-test using GraphPad Prism 5.00 software.

### Therapeutic Efficacy

The HT-29 tumor model was used to determine therapeutic efficacy. HT-29 cells (5 x 10^6^ cells in 0.05 mL media) were injected subcutaneously (s.c.) into the central lower backs of female Rag2-M mice (6–9 weeks old). Tumors appeared within 2 weeks following cell inoculation, and, at this time, mice were randomly separated into treatment groups of 6 mice per group, unless otherwise indicated. Treatments were initiated when tumors had reached an average volume of ∼150 mm^3^ (0.5–0.7 cm in diameter), which occurred around day 14 post-cell inoculation. Treatments were administered to mice as follows: saline+D5W, 5-FU (16 mg/kg), IRI (60 mg/kg), IrC™ (40 or 60 mg IRI/kg), IRI +5-FU (60 mg/kg +16 mg/kg), or IrC™ +5-FU (40 or 60 mg IRI/kg +16 mg/kg). D5W and 5-FU were administered daily for 5 days (QD×5) each week for 3 weeks via i.p. injection; all other treatments were administered once per week for 3 weeks (Q7D×3) via i.v. injection into the lateral tail vein. When mice received two different agents, either IRI and 5-FU or IrC™ and 5-FU, on the same day, 5-FU was injected at 2 h prior to IRI or IrC™ administration. In order to increase the total 5-FU exposure time, daily dosing of 5-FU was employed [39], based on the regimen described by Saltz *et al*. [40], [41], and the 16 mg/kg dose was selected after a dose escalation study determined that it was the MTD in Rag2-M mice (unpublished results). The highest IrC™ dose (60 mg IRI/kg) utilized in this study is well tolerated and is approximately 66% of the MTD determined by our group for Rag2-M mice. Intersecting tumor dimensions were measured 3 times per week; tumor volume was calculated using (ab^2^)/2 (a, larger dimension; b, smaller dimension). Mice were euthanized if body weight loss (BWL) exceeded 20%, if tumor volume exceeded 1000 mm^3^, if tumor ulceration was observed, or if significant deteriorations were observed in mouse health (clinical score as defined by an approved standard operating procedure). When assessing fold tumor volume increase, the tumor size on day 0 (day of treatment initiation) was defined as 1.

## Results

### 5-FU and IRI Show Exposure Time Dependency as Single Agents and in Combination

**Figure 1:**
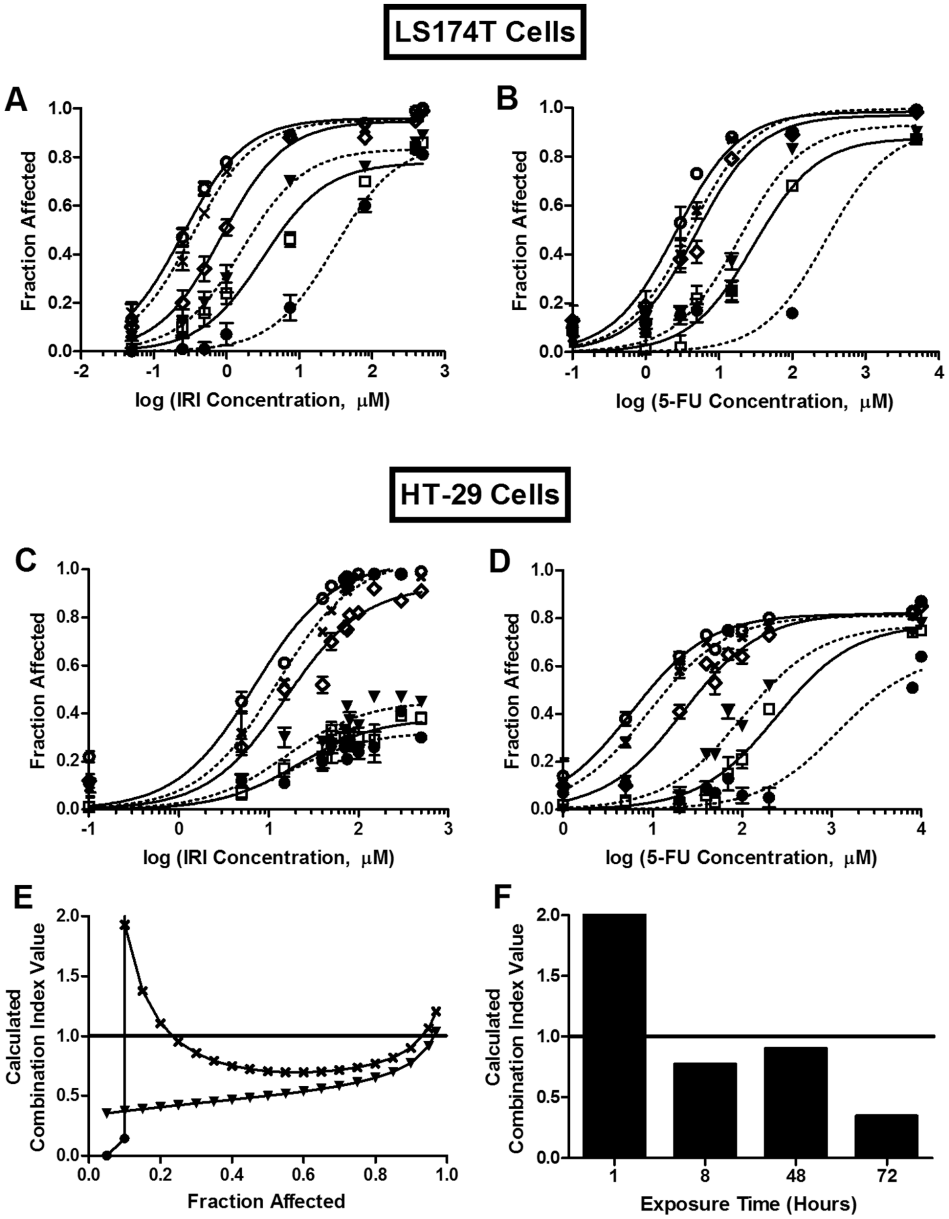
Exposure time dependency of IRI and/or 5-FU cytotoxicity *in vitro*. A–D) Single agent exposure time dependency. LS174T (A and B) and HT-29 (C and D) cells were exposed to IRI (A and C) or 5-FU (B and D) for 1 (•, dotted line), 4 (**□**, solid line), 8 (▾, dotted line) 24 (**⋄**, solid line), 48 (**X**, dotted line), or 72 h (○, solid line). E) Combination exposure time dependency. HT-29 cells were exposed to IRI/5-FU (1∶1 molar ratio) for 1 h (•), 8 h (▾), or 48 h (**X**). F) Calculated CI values at FA = 0.9 for HT-29 cells exposed to IRI/5-FU (1∶1 molar ratio) for 1–72 h. A–D) Each point represents the mean +/− standard deviation (n = 3–9) from 2–3 experiments, each completed in triplicate. E, F) Each point or bar represents a combination index value calculated from cytotoxicity data compiled from 2–4 separate experiments, each completed in triplicate. CI of 0.8 to 1.2 suggests additive interactions; CI <0.8 suggests synergistic interactions; and CI >1.2 suggests antagonistic interactions.

Drug combination effects are dependent on a number of factors, including drug-drug ratios, the orders and time sequences of administration, and exposure times. Previous studies have shown a drug ratio dependency for IRI/floxuridine combinations [42]; a similar drug ratio dependency was also shown in the current studies with combinations of IRI/5-FU (data not shown). However, the studies described here additionally considered the role of drug exposure times on drug combination effects. This might be achieved, for example, with the use of drug infusions, an optimized drug administration schedule, or through the use of nanoparticle anti-cancer drug formulations designed for optimized drug release rates and enhanced drug exposure times. The effect of exposure time on cytotoxicity/cytostasis was determined for combinations of IRI/5-FU, and these data are summarized in Fig. 1. The therapeutic effects of 5-FU and IRI, when used alone, were highly dependent on exposure time (Fig. 1A–D). For the LS174T cell line, the IC_50_ for IRI (0.3 µM; Fig. 1A) and 5-FU (3.0 µM; Fig. 1B) were over 100-fold lower when the drug exposure time was 72 h, relative to an exposure time of 1 h (44 µM and 330 µM, respectively). For the HT-29 cells, the IC_50_ for 5-FU was 9 µM for a drug exposure time of 72 h, compared to 4000 µM for an exposure time of 1 h (Fig. 1D). Further, the IC_50_ for IRI was not measurable in the HT-29 cells when the exposure time was 1, 4, or 8 h (Fig. 1C). It should be noted (see Methods) that in these studies, the toxicity assessments were determined at 72 h, and thus only the drug exposure time was varied here.

The cytotoxic effects of combinations of IRI/5-FU were determined for HT-29 cells for different exposure times. Short exposure times (1 h) produced strong antagonism, with CI values of greater than 5 at FA values of greater than 0.1 (Fig. 1E). In contrast, synergism (CI values less than 0.8) was observed over a broad range of FA values when the exposure time was increased to 48 h. At an FA value of 0.9 (i.e., the alamarBlue assay indicated a value that was 90% lower than that detected for the drug-free controls), the CI values were >10, 0.8, 0.9, and 0.4 when the exposure times were 1, 8, 48, and 72 h, respectively (Fig. 1F). These results suggest that exposure time is an important variable to consider when trying to measure drug-drug interactions in cell-based screening assays.

### PK/BD Studies Following Administration of 5-FU and IrC™ Alone and in Combination

**Figure 2:**
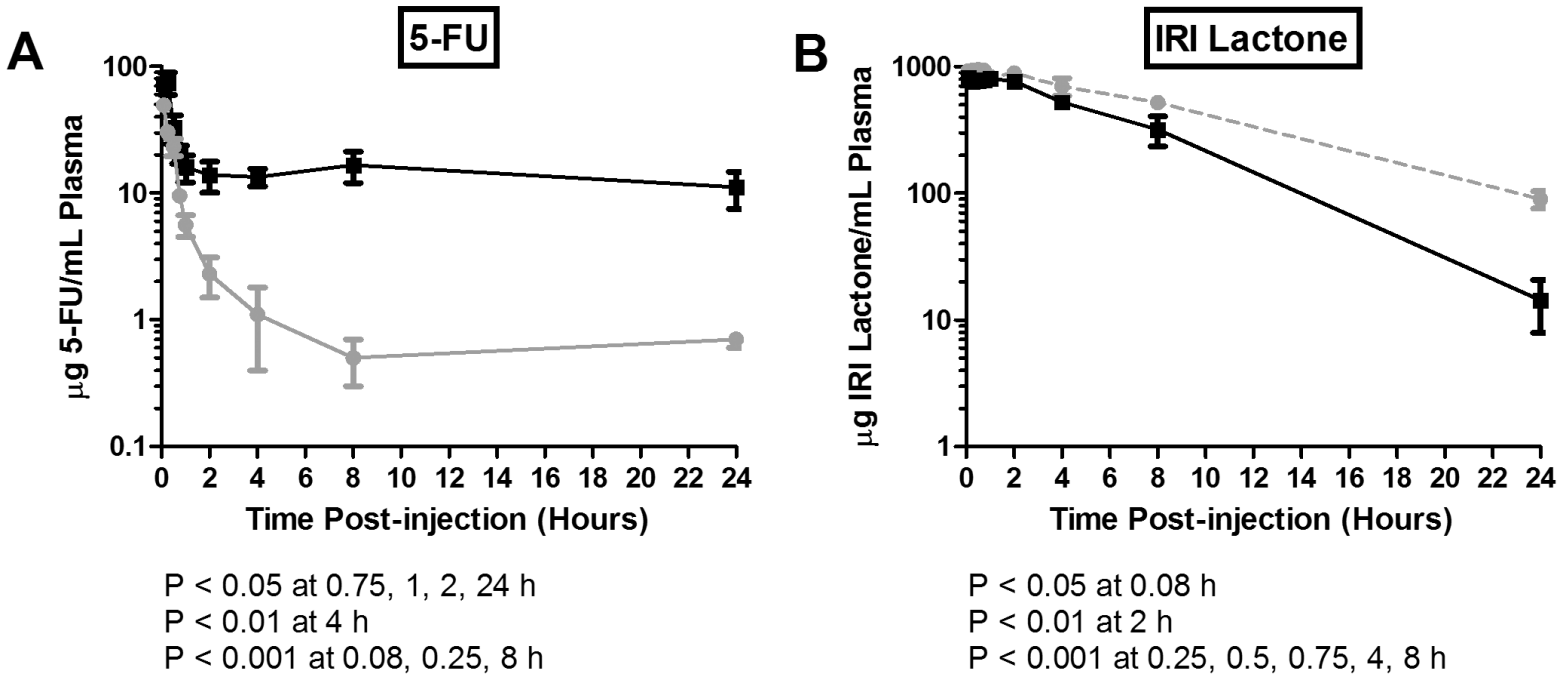
Plasma clearance of 5-FU and IrC™ administered as single agents or co-administered. Mice were injected i.v. with radio-labeled 5-FU (40 mg/kg) or IrC™ (40 mg IRI/kg), or both agents simultaneously. At various time points post-injection, the plasma concentrations of 5-FU and IRI (lactone) were determined. A) Mean plasma concentration of 5-FU +/− standard deviation (n = 4) after administration alone (solid gray line) or after co-administration with IrC™ (solid black line). B) Mean plasma concentration of IRI lactone +/− standard deviation (n = 4) after administration of IrC™ alone (dashed gray line) or after co-administration of IrC™ with 5-FU (solid black line).

**Figure 3:**
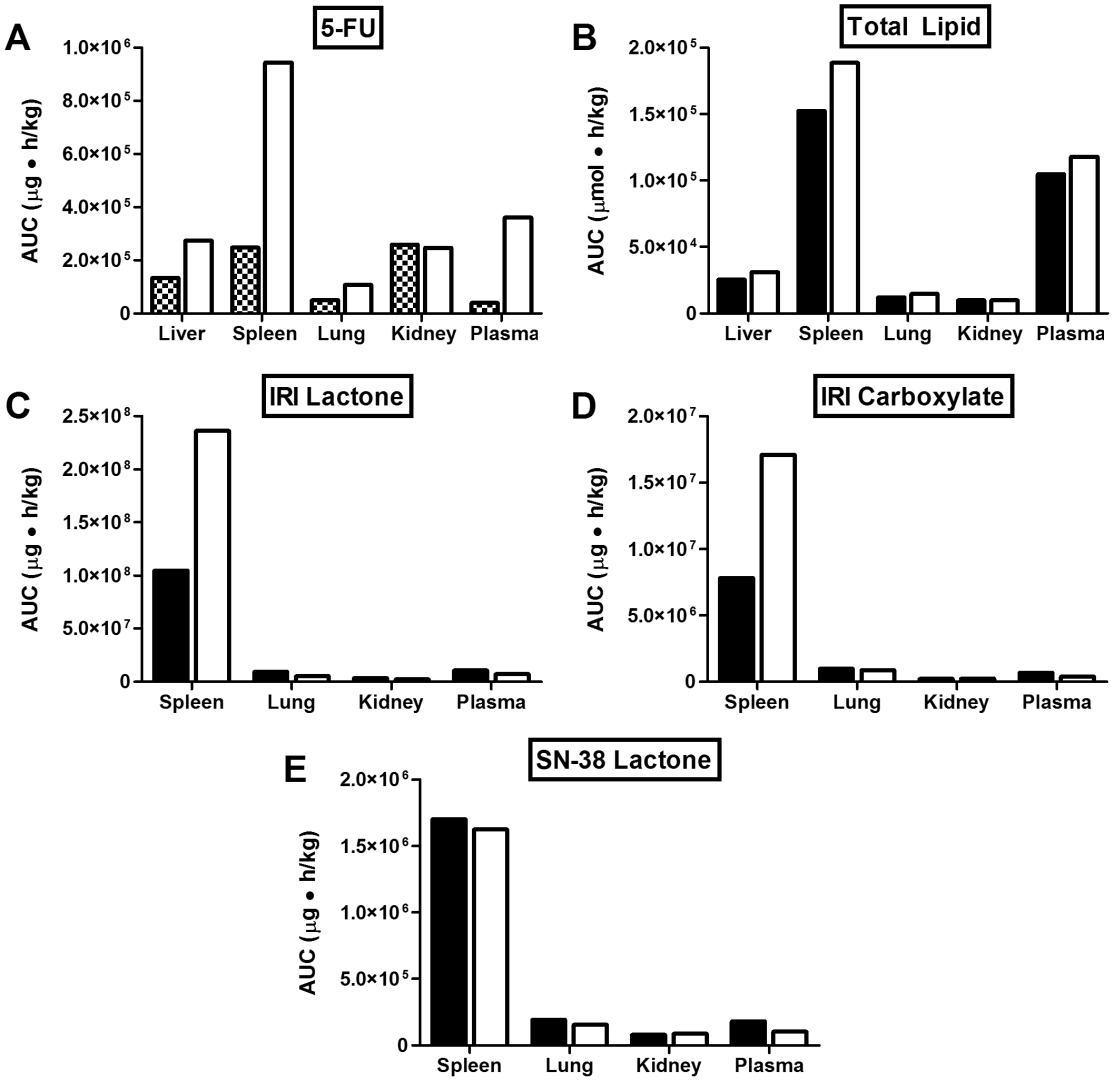
Mean AUC_0–24h_ of 5-FU and IrC™ administered i.v. as single agents or co-administered. Mice were injected i.v. with radio-labeled 5-FU (40 mg/kg; hatched bars) or IrC™ (40 mg IRI/kg; black bars), or both agents simultaneously (white bars). At various time points post-injection, the plasma and organ concentrations of the lipid and drug species were determined, and AUC_0–24h_ values were calculated from the resulting concentration-time curves. Data are presented as mean plasma and organ area under the curve (0–24 h) (n = 4) for 5-FU (A), total lipid (B), IRI lactone (C), IRI carboxylate (D), and SN-38 lactone (E).

**Figure 4:**
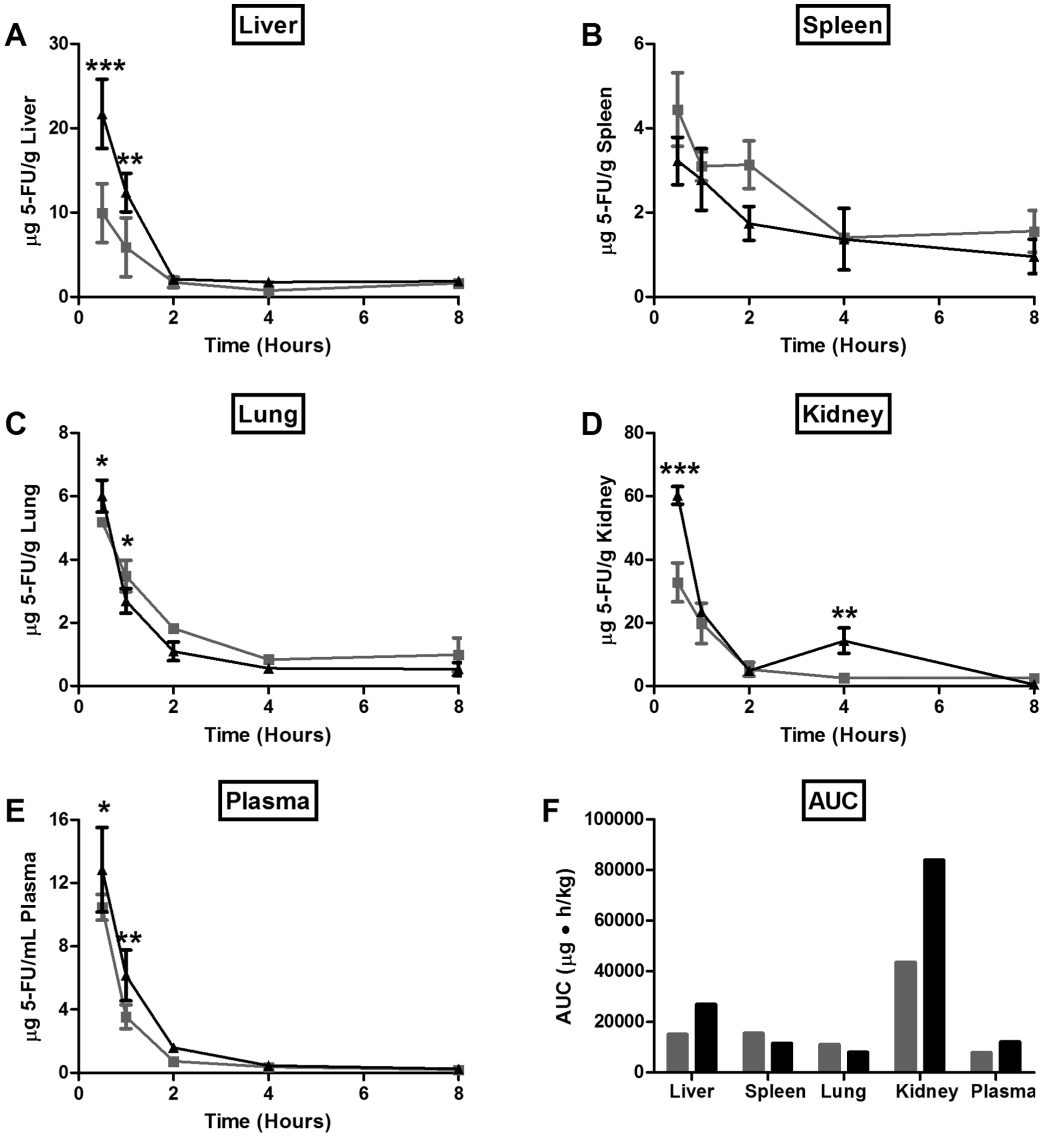
PK/BD of 5-FU administered i.p. as a single agent or co-administered with IrC™. Mice were injected i.p. with 5-FU (16 mg/kg) on days 1 and 2 (gray line/bar); 5-FU was spiked with radio-labeled 5-FU on day 2. Half of the mice were also injected i.v. with IrC™ (60 mg IRI/kg) on day 1 (black line/bar), at 2 hours after the injection of 5-FU. At various time points post-injection, the plasma and tissue concentrations of 5-FU were determined, and AUC_0–8h_ values were calculated from the resulting concentration-time curves. Data are presented as mean concentration of 5-FU in liver (A), spleen (B), lung (C), kidney (D), plasma (E) +/− standard deviation (n = 3), or mean plasma and organ area under the curve (0–8 h) (n = 3) for 5-FU (F).

For *in vivo* studies assessing the combination effects of IrC™ with 5-FU, it is important to first determine if one of the drugs alters the pharmacokinetics or biodistribution behavior of the other drug when they are co-administered (Fig. 2–4). The plasma elimination curves for 5-FU administered alone or in combination with IrC™ are summarized in Fig. 2A. When administered alone, 5-FU was rapidly cleared and the plasma levels of 5-FU were less than 3% of the injected dose within 15 min of injection. The plasma AUC_0–24h_ for 5-FU, co-administered with IrC™, was almost 10-fold higher than that seen for 5-FU administered alone, a result that is most evident at time points beyond 1 h (Fig. 3A). The higher plasma 5-FU levels were also associated with higher levels of 5-FU in the liver, spleen, and lungs, but not the kidneys (Fig. 3A). The results suggest that 5-FU elimination was reduced when the drug was co-administered (i.v.) with IrC™.

The plasma elimination curves for IRI lactone following the administration of IrC™ alone, or in combination with 5-FU, are shown in Fig. 2B. At the early time points up to 4 h post-injection, there was a small, but significant, decrease in the plasma levels of IRI lactone for animals injected with IrC™ in combination with 5-FU, versus IrC™ alone; at the 8 and 24 h time points, a more pronounced decrease in plasma IRI lactone levels was observed for mice that were co-administered IrC™/5-FU, compared to single agent IrC™. However, when assessing the plasma AUC_0–24h_ data calculated for liposomal lipid (Fig. 3B), IRI in the lactone form (Fig. 3C) or carboxylate form (Fig. 3D), and SN-38 in the lactone form (Fig. 3E) the values were essentially equivalent to the plasma AUC_0–24h_ determined following the administration of IrC™ alone. This was also reflected in the tissue AUC_0–24h_ data (Fig. 3B–E), with the exception of the spleen, where elevated levels of IRI lactone and IRI carboxylate were observed following co-administration (i.v.) of IrC™/5-FU, relative to IrC™ alone. HPLC analysis of IRI and SN-38 in the liver could not be performed in animals given IrC™ or IrC™/5-FU, due to spectral interference from an unknown molecule that was co-extracted with IRI and SN-38 from the liver homogenate. Although the assay used in these studies was capable of detecting SN-38 in the carboxylate form, it was not detected in any plasma or tissue samples above the HPLC limit of detection of 10 ng/mL.

The efficacy studies described below assessed the activity of 5-FU (alone and in combination) using a dose intense (daily) schedule, a schedule which made it necessary to administer the drug intraperitoneally. The change in PK/BD noted above, when the drugs were both given intravenously, was also expected to be less of a concern when IrC™ was given i.v. and 5-FU was given i.p. The results presented in Fig. 4 address this study design. Consistent with the results in Fig. 3, the data suggest, at least at the early time points, that the plasma and organ concentrations of 5-FU are higher when IrC™ is administered in combination with 5-FU. This effect was less evident than when dosing both drugs i.v., as the 5-FU concentrations were not statistically different at most time points beyond 2 h. However, at 0.5 h post-injection, the 5-FU concentrations were higher in the liver (P<0.001; Fig. 4A), lung (P<0.05; Fig. 4C), kidney (P<0.001; Fig. 4D), and plasma (P<0.05; Fig. 4E) of mice that were given IrC™/5-FU, relative to those animals that were given 5-FU alone. Compared to animals injected with 5-FU as a single agent, when mice received the combination of IrC™/5-FU, 2-fold higher concentrations of 5-FU were detected in the liver at 0.5 h (P<0.001) and 1 h (P<0.01) post-injection, and a corresponding increase in the liver AUC_0–8h_ of 5-FU (Fig. 4F) was also observed. The AUC_0–8h_ of 5-FU in plasma (Fig. 4F) was ∼1.5-fold higher following the administration of IrC™/5-FU, when compared to animals given 5-FU alone. A substantial increase in the AUC_0–8h_ of 5-FU in kidney (Fig. 4F) was observed when animals received an i.v. dose of IrC™, compared to mice given 5-FU alone.

### Efficacy of 5-FU and IrC™ Alone and in Combination

**Figure 5:**
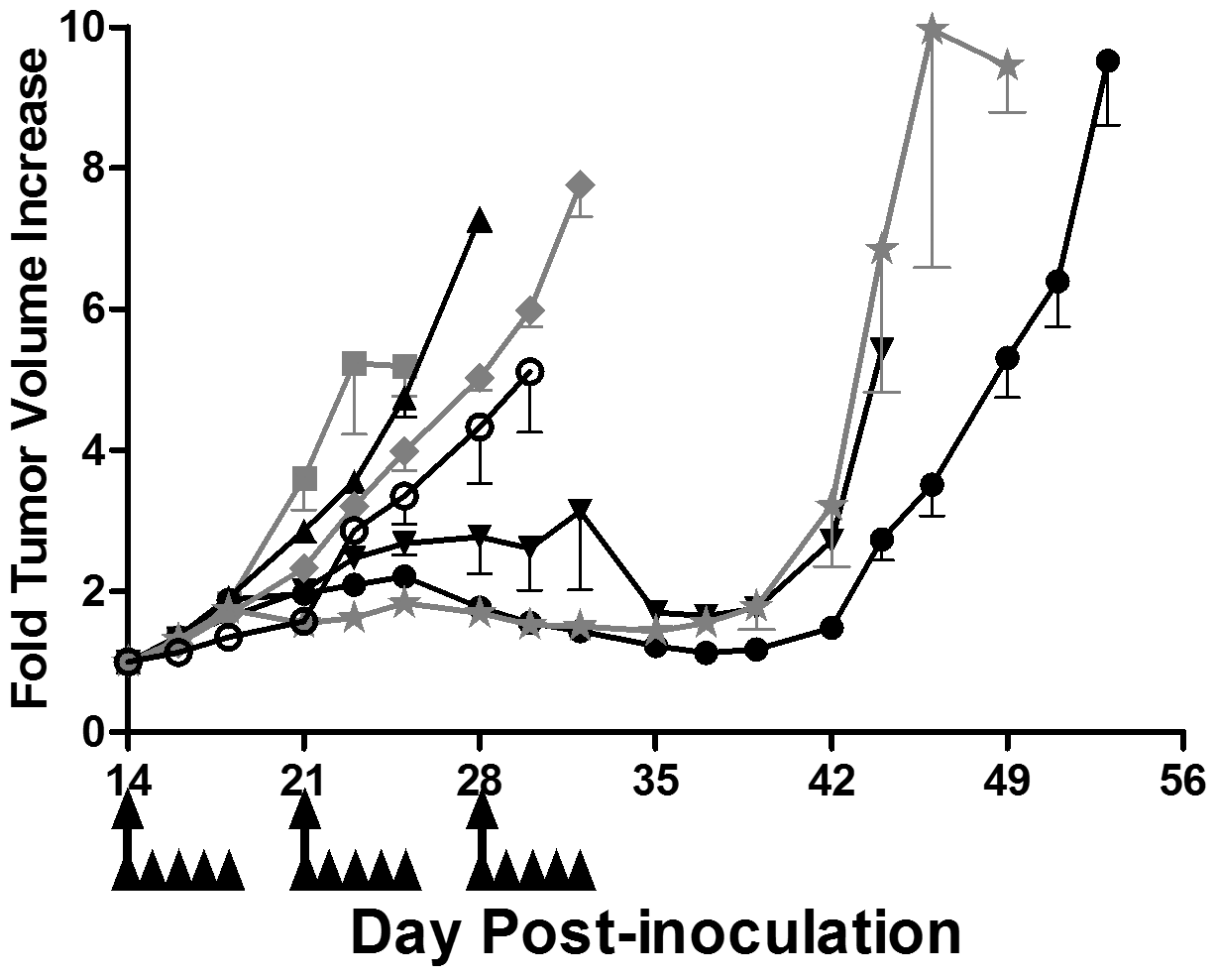
Efficacy of IRI/5-FU and IrC™/5-FU treatment in the HT-29 s.c. model of CRC. Mice bearing s.c. HT-29 tumors were treated with saline+D5W (grey solid square), 5-FU (16 mg/kg; black solid upright triangle), IRI (60 mg/kg; grey solid diamond), IrC™ (40 or 60 mg IRI/kg; black solid inverted triangle or black solid circle, respectively), IRI +5-FU (60 mg/kg +16 mg/kg; black open circle), or IrC™ +5-FU (40 mg IRI/kg +16 mg/kg; grey solid star). Beginning on day 14, D5W and 5-FU were administered QD×5 (x 3 weeks) via i.p. injection (arrowheads); all other treatments were administered Q7D×3 via i.v. injection (full arrows). Data are presented as mean fold tumor volume increase +/− standard error of the mean (n = 6).

The results of mouse studies assessing the efficacy of IRI and IrC™, with and without 5-FU, for the treatment of CRC are presented in Fig. 5. Maximum mean BWL was used as a measure of therapy-induced toxicity following treatment, and these data have also been summarized in Table 1. Free IRI was dosed at 60 mg/kg, which is the highest dose of free IRI that could be administered Q7D×3 to Rag2-M mice without engendering greater than a 10% mean BWL, in addition to other changes in animal health status. The 5-FU dose of 16 mg/kg, administered QD×5 each week for 3 weeks, was the maximum tolerated dose consistent with a maximum mean BWL of ∼10%. As illustrated in Fig. 5, when used alone at this dose, 5-FU exhibited little therapeutic activity in this model. The time to reach a 5-fold increase in tumor volume was 23 days for control mice and 26 days for mice treated with 5-FU, a tumor growth delay of only 13%. The therapeutic benefits of free IRI given at 60 mg/kg were not substantially better; the time to reach a 5-fold increase in tumor size was 28 days (a growth delay of 22%). Single agent IrC™ (60 mg IRI/kg) exhibited substantial therapeutic effects, with some tumor regression noted shortly after the last treatment. The time to reach a 5-fold increase in tumor size was 49 days, a 113% tumor growth delay when compared to control.

**Table 1:** Efficacy and toxicity of IRI, IrC™, and 5-FU administered as single agents and in combination.

Treatment	5-fold Tumor Volume Increase (Days)	Tumor Growth Delay vs. Control (%)	Mean Maximum BWL (+/− SEM) (%)
D5W+Saline	23	0	4.7+/−0.3
5-FU (16 mg/kg)^a^	26	13.0	9.1+/−3.4
IRI (60 mg/kg)^b^	28	21.7	7.0+/−1.8
IrC™ (40 mg IRI/kg)^b^	44	91.3	6.9+/−2.6
IrC™ (60 mg IRI/kg)^b^	49	113.0	6.1+/−2.1
IRI +5-FU (60+16 mg/kg)a,b	30	30.4	15.7+/−2.9
IrC™ +5-FU (40 mg IRI/kg +16 mg/kg)a,b	43	87.0	14.8+/−1.2
IrC™ +5-FU (60 mg IRI/kg +16 mg/kg)a,b	N/A*	N/A*	21.4+/−1.3

* Unable to determine as treatment group euthanized early due to excessive BWL.

a Beginning on day 14 post-implantation, 5-FU was administered QD×5 for 3 weeks.

b Beginning on day 14 post-implantation, IRI and IrC™ were administered Q7D×3.

When 5-FU (16 mg/kg) was combined with free IRI (60 mg/kg), there was a small, but not significant, improvement in therapeutic effect when compared to the effects of each drug used alone. The time to reach a 5-fold increase in tumor size was 30 days, compared to 23 days for control and 28 days for animals treated with IRI alone. The combination of IRI/5-FU resulted in an increase in toxicity that could be considered additive based on the effects of the agents when used alone. A maximum mean BWL of 16% was observed in animals treated with the combination. When used alone, each agent caused a maximum mean BWL of approximately 7% (IRI) or 9% (5-FU). When 5-FU (16 mg/kg) was combined with IrC™ (60 mg IRI/kg), a surprising increase in toxicity was observed. These animals showed dramatic weight loss (>20%) over the first week of dosing, and were euthanized prior to the start of the second treatment cycle. IrC™, when given as a single agent at 60 mg IRI/kg, resulted in a mean BWL of 6.1% (Table 1). Due to increases in toxicity, the dose of IrC™ in the combination treatment was reduced to 40 mg IRI/kg. Treatment with IrC™ (40 mg IRI/kg) alone resulted in a 91% tumor growth delay relative to control (a 5-fold increase in tumor volume by day 44). The toxicity at this dose, as judged by mean BWL, was comparable to that seen at the 60 mg IRI/kg dose (Table 1). Mice treated with the combination of 5-FU and IrC™ (40 mg IRI/kg) still showed an increase in toxicity (maximum mean BWL of 15.7%), but this dose was tolerated and allowed assessments of therapeutic activity. No further improvements in anti-tumor effects were observed when 40 mg IRI/kg IrC™ was combined with 5-FU. A 5-fold increase in tumor volume was observed on day 43. When compared to the equivalent dose of single agent IrC™ (40 mg IRI/kg), the same 5-fold increase was noted on day 44. Even with evidence suggesting that the combination resulted in increased toxicity, no gains in therapeutic activity were noted.

## Discussion

Evidence for the efficacy of IrC™ comes from a number of previous studies from our laboratory, which have demonstrated the significant therapeutic benefits of IrC™, at doses that were 3- to 5-fold lower than the MTD of free IRI (60 mg/kg when given i.v. Q7D×3 in Rag2-M mice (unpublished data)). These results were confirmed here using the HT-29 model of CRC, where single agent IrC™, dosed at 40 or 60 mg IRI/kg, was well tolerated (causing less than 7% maximum mean BWL) and resulted in significant delays in tumor growth, including tumor regression at the higher dose. The objectives of the current studies, however, were to establish the therapeutic potential of prolonged exposure to IRI and 5-FU. This was assessed *in vitro* through evaluations of the cytotoxicity of free IRI and 5-FU, alone and in combination. Therapeutic activity was also measured *in vivo*, following treatment with IrC™ in combination with 5-FU administered via a dose-intensive daily schedule.

As noted above, the cytotoxicity assays were completed with free IRI to assess how the duration of IRI exposure affects its activity when used alone and in combination with 5-FU. Prolonged exposure to free IRI has been used to mimic the exposure to IRI achieved when administering the drug *in vivo*, in a well-designed drug carrier formulation, such as IrC™. The *in vitro* results presented here (Fig. 1) show strong time-dependent cytotoxicity for both IRI and 5-FU against the target tumor cell population, and proved the synergistic activity of both drugs in combination, particularly when the drug exposure time was lengthened. These data helped to justify the *in vivo* investigations assessing the therapeutic potential of a combination of a sustained-release formulation of IRI (IrC™) and 5-FU given via a dose-intense schedule (daily dosing). It could be argued that the ideal combination arising from the *in vitro* studies would include a combination of a sustained-release formulation of 5-FU with IrC™. Our lab has been developing a liposomal formulation of 5-FU [43] to pursue these studies in the future.

The studies summarized here are the first to assess the therapeutic potential of 5-FU combined with IrC™. Nakajima *et al*. [22] have demonstrated therapeutic success in the treatment of HT-29 tumors when administering free 5-FU in combination with a micellar formulation of SN-38. These authors attribute the improved therapeutic effect of the combination (relative to free SN-38 and 5-FU) to prolonged drug exposure achieved when SN-38 is delivered via a polymeric formulation [22]. Previous research from our lab has shown that animals that have been treated with IrC™ maintain SN-38 levels in the plasma compartment for extended time periods [21], and for this reason, it was reasonable to expect that combinations of IrC™ and a dose-intense schedule of 5-FU would result in significant benefits.

Despite the clinical utility of the combination of IRI/5-FU [44], and the expectations of strong therapeutic activity for IrC™/5-FU, our results convincingly demonstrated that the addition of 5-FU to IrC™ monotherapy provided no further benefit in the subcutaneous HT-29 model in mice. Increasing the exposure time of 5-FU has been shown by others to increase its efficacy [45], [46]. However, the dose-intense schedule of 5-FU, while efficacious, resulted in only modest activity in the HT-29 model. In the clinic, systemic dosing of 5-FU via infusion is often preferred over i.v. bolus administration [45]. Infusions can mitigate toxicities associated with the peak plasma concentrations of 5-FU [47], and, more specifically, reduce the accumulation of 5-FU in bone marrow [48]. Interestingly, a number of recent clinical trials have investigated the therapeutic potential of post-operative biweekly i.p. injections of 5-FU in combination with systemically administered chemotherapy [49], [50]. This type of adjunct 5-FU treatment has demonstrated good success. For example, Vaillant *et al*. [51] reported that treatment of post-operative stage II CRC patients with daily injections of i.p. 5-FU for 6 days, and no other chemotherapy, led to an increase in 5 year disease-free survival rates.

There are several possible explanations as to why there were no therapeutic benefits observed when using IrC™ in combination with daily injections of i.p. 5-FU. First, the choice of the HT-29 tumor model for *in vivo* studies may not have been ideal, as this model was reasonably insensitive to 5-FU, even when the drug was administered via a dose-intense daily schedule. It has been reported that the level of thymidylate synthase (TS) in tumor tissue may be correlated with response to 5-FU therapy [52]; thus, future studies should include careful consideration of the TS levels of different tumor models prior to experimentation. Another factor that should be considered here is the possibility of camptothecin-mediated down-regulation of dihydropyrimidine dehydrogenase (DPD) [53]. DPD is the enzyme primarily responsible for catabolising/detoxifying 5-FU [54]. Down-regulation of DPD can lead to fatal toxicities following 5-FU treatment, as has been observed clinically with DPD-deficient patients [55]. In addition, researchers have shown that treatment with SN-38 [56] or IRI [57] can lead to inhibition of TS, the target enzyme of 5-FU, thereby increasing cellular sensitivity to 5-FU and increasing the likelihood for toxicity. There is conflicting evidence about the effect of 5-FU on the metabolism of SN-38, with some studies showing no interaction between the drugs [58], and others suggesting that 5-FU may decrease the AUC of SN-38 [59] – although this may be dependent on the 5-FU dosing regimen [59] and/or the formulation used to enhance SN-38 exposure. At present, the interaction between the two drugs is not well understood.

Second, as highlighted by the results summarized in Fig. 2–4, the co-administration of IrC™/5-FU changed the PK/BD properties of 5-FU, relative to the PK/BD of 5-FU when it was administered i.v. or i.p. as a single agent. Data revealed that, when given simultaneously with i.v. 5-FU, IrC™ engenders significant decreases in 5-FU elimination (Fig. 2A) and an associated increase in 5-FU plasma AUC_0–24h_ (Fig. 3A). Similarly, when mice were treated with i.p. 5-FU (QD), with and without a single i.v. dose of IrC™ at 60 mg IRI/kg on day 1, there were significant changes in the PK/BD properties of 5-FU (Fig. 4). Specifically, statistically significant increases in peak concentrations of 5-FU were observed in the plasma, kidney, and liver at 0.5 and/or 1 h post-injection, when 5-FU was co-administered with IrC™ at 60 mg IRI/kg when compared to mice who were administered 5-FU as a single agent. Associated changes in plasma and organ AUC_0–8h_ values suggest that a drug-drug interaction occurs in mice when i.p. 5-FU is administered with IrC™. It is not clear if similar increases in the 5-FU plasma AUC would be observed in a clinical setting following co-administration of IrC™/5-FU.

Changes in the PK/BD of 5-FU are significant when considering the toxicity observed in the studies summarized here. Both 5-FU and IRI are known to be GI toxic agents [15], [16], [60], [61], [62]. GI toxicity is typically associated with BWL in mice and, unexpectedly, the combination of 5-FU (16 mg/kg) and IrC™ (60 mg IRI/kg) caused significant weight loss in the Rag2-M mice, even though the administered doses were well tolerated as monotherapies (see Table 1), suggestive of a synergistic toxicity. Clinical studies have noted increased adverse toxicities when employing dosing regimens where IRI is administered after 5-FU treatment [59]; thus, one could anticipate that the toxicities observed here may be mitigated by administering the two drugs in a different sequence. Importantly, when considering the mechanism of activity of these two drugs, changing the sequencing of when the drugs are administered may eliminate the drug-drug interactions that caused changes in PK/BD. This may in turn reduce toxicity while also enhancing therapeutic outcomes. Researchers have shown that the IRI treatment can cause an increase in the percentage of tumor cells in S-phase [56], [63]. This, in turn, would increase the number of cells susceptible to the actions of 5-FU, which causes DNA damage to cells in S-phase following prolonged exposure. It has been suggested that this interaction between the effects of the drugs contributes to the synergistic efficacy observed in some tumor models when 5-FU and IRI were administered sequentially (IRI administered prior to 5-FU) [39], [56], [57], [63], [64]. Although these studies are beyond the scope of the objectives presented here, it will be important in future investigations to examine the effects of drug sequencing/timing on cell cycle progression to improve and optimize the therapeutic results for the combination of IrC™/5-FU treatment.

Related to this point, it has been shown that IrC™ treatment can cause changes in tumor-associated blood vessels that are comparable to those observed when using anti-angiogenic drugs [29], [31]. These changes have been described in the context of vascular normalization and it can be further suggested that vascular normalization may promote enhanced delivery of small molecular weight drugs, such as 5-FU [31], [65]. Thus, it could be anticipated that 5-FU should be administered after IrC™ has achieved normalization of tumor vasculature. This type of administration schedule is currently being tested in our lab. Further investigations of IRI/5-FU or IrC™/5-FU combinations should also explore the use of 5-FU dosed in sustained-release formulations [43], [66] or sustained-release formulations that contain both IRI and 5-FU at a fixed dose ratio [67], [68]. Both of these dosing options may potentially have the advantage of making it possible to achieve synergistic drug ratios for an extended period of time at the target site [67], [68], [69], while minimizing exposure in sites of potential toxicities. This strategy has been shown to be important for producing synergistic anti-cancer effects with IRI/floxuridine [42] and other drug combinations [70],[71]. Finally, it may be prudent to explore the use of leucovorin to potentiate the efficacy of 5-FU [72], [73] in the HT-29 model.

### Conclusions

The importance of achieving prolonged exposure times to 5-FU and IRI, when used alone and in combination, was demonstrated through a series of *in vitro* cytotoxicity experiments evaluating drug synergies as a function of exposure time. *In vivo* studies revealed that 5-FU and IrC™, when co-administered, caused significant changes in the PK/BD profile of 5-FU. Efficacy studies in a murine xenograft model of human CRC showed that single agent IrC™ was significantly more efficacious than the combination of free IRI/5-FU. Use of IrC™ alone resulted in a higher therapeutic index than the combination of IrC™/5-FU, which caused significant increases in toxicities. Enhanced toxicity was likely due to IrC™-engendered changes in the PK/BD of 5-FU. If 5-FU is to be used in combination with IrC™, then studies exploring how efficacy and toxicity are influenced by different dose sequences need to be completed.
